# Association between adverse events after COVID-19 vaccination and anti-SARS-CoV-2 antibody concentrations, the Netherlands, May 2021 to November 2022: a population-based prospective cohort study

**DOI:** 10.2807/1560-7917.ES.2024.29.25.2300585

**Published:** 2024-06-20

**Authors:** Minke R Holwerda, Christina E Hoeve, Anne J Huiberts, Gerco den Hartog, Hester E de Melker, Susan van den Hof, Mirjam J Knol

**Affiliations:** 1 Centre for Infectious Disease Control, National Institute for Public Health and the Environment, Bilthoven, the Netherlands

**Keywords:** SARS-CoV-2, COVID-19 vaccination, adverse events, serology, antibody response

## Abstract

**Background:**

Non-severe adverse events (AE) including pain at injection site or fever are common after COVID-19 vaccination.

**Aim:**

To describe determinants of AE after COVID-19 vaccination and investigate the association between AE and pre- and post-vaccination antibody concentrations.

**Methods:**

Participants of an ongoing prospective cohort study (VASCO) completed a questionnaire on AE within 2 months after vaccination and provided 6 monthly serum samples during May 2021–November 2022. Logistic regression analyses were performed to investigate AE determinants after mRNA vaccination, including pre-vaccination Ig antibody concentrations against the SARS-CoV-2 spike protein receptor binding domain. Multivariable linear regression was performed in SARS-CoV-2-naive participants to assess the association between AE and log-transformed antibody concentrations 3–8 weeks after mRNA vaccination.

**Results:**

We received 47,947 completed AE questionnaires by 28,032 participants. In 42% and 34% of questionnaires, injection site and systemic AE were reported, respectively. In 2.2% of questionnaires, participants sought medical attention. AE were reported more frequently by women, younger participants (< 60 years), participants with medical risk conditions and Spikevax recipients (vs Comirnaty). Higher pre-vaccination antibody concentrations were associated with higher incidence of systemic AE after the second and third dose, but not with injection site AE or AE for which medical attention was sought. Any AE after the third dose was associated with higher post-vaccination antibody concentrations (geometric mean concentration ratio: 1.38; 95% CI: 1.23–1.54).

**Conclusions:**

Our study suggests that high pre-vaccination antibody levels are associated with AE, and experiencing AE may be a marker for higher antibody response to vaccination.

**Key public health message**

**What did you want to address in this study and why?**

Adverse events (AE) such as pain at injection site or fever are common after COVID-19 vaccination. There may be a relationship between AE after COVID-19 vaccination and the body’s immune response. We wanted to understand the factors causing AE after COVID-19 vaccination and to investigate the relationship between AE and antibody levels before and after primary and booster vaccination (mRNA vaccine doses 2–4) in the general population.

**What have we learnt from this study?**

Surveying over 28,000 people, 42% reported injection site AE and 34% reported systemic AE. Adverse events were generally mild and more often reported by women, people under 60 years, those with medical risk conditions and who received the Spikevax vaccine (vs Comirnaty). Having higher antibody levels before vaccination was associated with systemic AE after doses 2 and 3. Any AE after dose 3 was associated with higher antibody levels.

**What are the implications of your findings for public health?**

Our study shows that AE are generally mild and suggests that having high antibody levels before vaccination is associated with AE, and that experiencing AE may be a marker for a good antibody response to mRNA vaccination. In other words, experiencing an AE is likely an indication that the body is developing protection after vaccination. Evidence for this association may increase acceptance of AE after vaccination.

## Introduction

On 11 March 2020, the World Health Organization (WHO) officially declared the severe acute respiratory coronavirus 2 (SARS-CoV-2) outbreak a pandemic [1]. By the end of 2020, vaccines for COVID-19 became available in Europe [2]. These vaccines showed good protection and acceptable safety profiles in clinical trials [3]. However, long-term protection and occurrence of adverse events (AE) in a real-world setting need to be monitored after licensure. Furthermore, additional data are required on the safety of COVID-19 vaccines in specific groups not included in clinical trials, such as people with comorbidities [4].

A Dutch study monitoring the outcomes following the first COVID-19 vaccine dose of over 20,000 individuals in the Netherlands found that almost two-thirds of people experienced at least one AE after vaccination [5]. An AE after vaccination includes any medical occurrence and does not necessitate a causal relationship between the AE and vaccination [6]. Despite this, AE following vaccination can lead to negative attitudes towards vaccination and vaccine refusal [7]. Yet, it is also commonly believed that AE are a positive sign as they can be indicative of the COVID-19 vaccine eliciting a good immune response [8,9]. Evidence for this association between AE and antibody response may increase acceptance of AE after vaccination. However, the few studies assessing the relationship between COVID-19 vaccine AE and antibody concentrations have produced mixed results [9,10].

Previous studies on the association between AE and antibody response after COVID-19 vaccination generally have small sample sizes, are performed in specific populations, e.g. healthcare professionals (HCP), and primarily include only the first and second Comirnaty (BNT162b2 mRNA, BioNTech-Pfizer) vaccine doses [9-15]. We use data on AE and antibody response after vaccination from the VAccine Study COvid-19 (VASCO), which included over 45,000 individuals from the general Dutch population. This study has collected information on vaccination with Vaxzevria (ChAdOx1 nCoV-19, Oxford-AstraZeneca), Spikevax (mRNA-1273, Moderna), Comirnaty and Jcovden (Ad26.COV2-S, Janssen-Cilag International NV), as well as information on specific groups such as those with comorbidities. To probe the relationship between AE and antibody response, we aimed to investigate determinants of solicited AE and AE for which medical care was sought after COVID-19 vaccination in the Netherlands and to determine the relationship between self-reported AE and anti-SARS-CoV-2 antibody concentrations before and after mRNA vaccination.

## Methods

### Study design 

This study was performed within VASCO, an ongoing population-based prospective cohort study with the primary objective to estimate vaccine effectiveness (VE) of COVID-19 vaccines used in the Netherlands [16]. Recruitment for VASCO was performed between 3 May 2021 and 15 December 2021 via social media as well as regular mail using a random sample of the national Dutch Personal Records Database, which contains a postal address for all individuals in the Netherlands. Community-dwelling Dutch adults aged 18 to 85 years who were able to read, understand and write Dutch were included. In VASCO, participants are more often women, highly educated and of Dutch ethnicity compared with the overall population [16].

Data are collected through digital questionnaires available to participants in a mobile app or website. Information on sociodemographic variables, health status, COVID-19 vaccination status and SARS-CoV-2 infection test results were collected via digital questionnaire at baseline, and thereafter once per month in the first year of participation and once per quarter in years 2 to 5. 

Participants were asked to notify in the app when they received a COVID-19 vaccination. A questionnaire on all AE (solicited and AE for which medical care was sought) was subsequently made available to participants 1 month after each COVID-19 vaccination. Serum to measure anti-SARS-CoV-2 antibody concentrations was obtained by a self-collected fingerprick blood sample at baseline (0), 6 and 12 months, and 1 month after primary vaccination series. See Supplementary File S1 for an overview of the different sampling moments.

The current analysis includes AE questionnaire data collected up to 14 November 2022 and serology data up to 6 November 2022. For a full description of the VASCO study, please see [16].

### Inclusion criteria

In this study, we included participants with a completed AE questionnaire within 2 months following vaccination. Additional in- and exclusion criteria were applied according to each research objective.

To investigate the relationship between AE and pre-vaccination anti-SARS-CoV-2 antibody concentrations, a serology sample within 8 weeks before vaccination was required. Participants were excluded if they reported a positive SARS-CoV-2 test between blood sampling and vaccination. To investigate the relationship between AE and post-vaccination anti-SARS-CoV-2 antibody concentrations, a serology sample within 8 weeks after vaccination was required, and participants were excluded if they had a history of SARS-CoV-2 infection before vaccination based on a self-reported positive SARS-CoV-2 test or serology results.

### COVID-19 vaccination schedule in the Netherlands

COVID-19 vaccination started on 6 January 2021, with Comirnaty, Spikevax, Vaxzevria and Jcovden. Vaxzevria was no longer used after November 2021. Nuvaxovid (NVX-CoV2601, Novavax) was offered from March 2022 onwards. Healthcare workers (HCW) and residents of long-term care facilities or institutions were targeted first. From 26 January 2021 onward, mobile persons in the general population were invited, in ascending order of birth year, to receive their vaccination. Medical risk groups were targeted from 17 March 2021 onward. Booster vaccination started on 18 November 2021, using primarily mRNA vaccines Comirnaty and Spikevax. JCovden was offered to those who did not want to or for medical reasons could not receive a booster mRNA vaccine [17]. Because of low numbers, Nuvaxovid vaccinations are not included in the analysis. 

### Data collection

#### Adverse events

The first version of the questionnaire on AE (used from May 2021 to August 2021) contained only two questions: (i) whether participants sought medical attention for an AE and (ii) if so, to specify which AE in an open text field. From August 2021 onwards, questions were added on the occurrence of overall injection site AE (e.g. pain, redness and swelling at the injection site or axillary region) and overall systemic AE (e.g. headache, myalgia, fatigue, nausea, diarrhoea and other symptoms of malaise), duration of AE (< 1 day, 1–2 days, 3–4 days, 5 or more days) and severity of AE (10-point Likert scale)*.* *The Likert score was used in a simple 10-point scale to indicate how severe the symptoms were to the participant, where 1 represented very mild symptoms, and 10 represented very severe symptoms. No specific validation study was done within VASCO considering the use of the Likert score.* All adverse events were self-reported and not medically confirmed. See Supplementary File S2 for the complete questionnaire.

Three types of participants were identified: those with only injection site AE, only systemic AE or both. The AE scores were determined by calculating the product of the duration and severity. For participants with both injection site and systemic AE, the sum of the AE scores was calculated. We used duration scores of 0.5, 1.5, 3.5 and 6 for the four duration categories. The AE scores of each group were then categorised as mild or moderate severity. Mild severity was defined as an AE score below or equal to the median score of the group; moderate severity was defined as an AE score above the median score of the group. Categorisation was performed by dose.

#### Antibody detection

Serum samples were tested using the Elecsys Anti-SARS-CoV-2 S and Anti-SARS-CoV-2 assays (Roche Diagnostics). These electrochemiluminescence immunoassays measure total Ig levels against the receptor binding domain (RBD) domain of the spike protein (S-antibodies) and the nucleoprotein (N-antibodies) of SARS-CoV-2 to distinguish between vaccine-induced antibodies (S-antibodies) and infection-induced antibodies (N-antibodies). Results are expressed in binding antibody units (BAU)/ml.

### Data analysis

#### Determinants of adverse events

Data were analysed separately for solicited AE and for AE for which individuals actively sought care. For solicited AE, the percentage of subjects who reported injection site AE and systemic AE were presented overall and stratified by age group (18–59 vs 60–85 years), gender (man, woman, other), medical risk group, vaccine product (Comirnaty, Jcovden, Spikevax, Vaxzevria), and vaccine dose (Doses 1–5). Participants were categorised as belonging to a medical risk group if they had one or more of the following conditions: asplenia, cancer (in the past, currently treated, currently untreated), cardiovascular disease, diabetes, immune disorder, kidney disease, liver disease, lung disease or asthma, neurological disease, organ or bone marrow transplantation. Pregnant women were not included as a medical risk group in this analysis.

The percentage of participants who sought medical care concerning a potential AE were presented overall and stratified by age group, gender, medical risk group, vaccine dose and vaccine product. The AE for which medical care was sought were categorised by the Netherlands Pharmacovigilance Centre Lareb (https://www.lareb.nl/en) using medical terminology dictionary for regulatory activities (MedDRA) higher level terms (HLT) and frequencies were presented. Potential determinants of AE occurrence were investigated using multivariable logistic regression analysis. Variables included in the model were age group, gender, medical risk group, vaccine product and vaccine dose. As data on JCovden and Vaxzevria vaccines were limited, this analysis was restricted to the mRNA vaccines (Comirnaty and Spikevax).

#### Association between pre-vaccination S-antibody concentrations and adverse events

We performed additional logistic regression analyses, stratified by dose, to investigate the association between pre-vaccination S-antibody concentrations (BAU/ml) and injection site AE, systemic AE and AE for which medical care was sought. Pre-vaccination S-antibody concentrations (BAU/ml) were categorised into quartiles, separately for each dose. We adjusted for time between blood sampling and vaccination (partly adjusted model) and additionally for age group, gender and vaccine product (fully adjusted model). Analysis was performed on participants who received an mRNA vaccine as Dose 2, 3 or 4, as there was limited data on Dose 1, since questions on solicited AE were included since August 2021.

#### Association between adverse events and post-vaccination S-antibody concentrations

Multivariable linear regression analysis was performed, stratified by dose, to assess the relationship between AE and post-vaccination log-transformed S-antibody concentrations. Covariates in each model were age group, gender, medical risk group, body mass index (BMI), vaccine product and time between blood sampling and vaccination. Three models were built with log-transformed S-antibody concentrations as the dependent variable and the following as independent variables: (i) the occurrence of any AE (basic model), (ii) the type of AE (injection site AE, systemic AE or both) (extended model) and (iii) the severity of AE (mild or moderate) per AE type (combined model). Analysis was restricted to participants having received a mRNA vaccine as Dose 2 or 3, and participants aged 60 years or older having received a mRNA vaccine as Dose 4. Data were limited for the younger age group (18–59 years) receiving Dose 4 as for this dose the vaccination policy in the Netherlands was to target people over 70 years of age, immunocompromised persons and adults with Down syndrome [17]. We present geometric mean concentration (GMC) ratios with 95% confidence intervals (CI).

Statistical analyses were performed in RStudio (v4.2.2) using the tidyverse (v1.3.2) and geepack (v1.3.9) packages [18-20].

## Results

A total of 47,947 unique AE questionnaires were completed by 28,032 participants between 10 May 2021 and 14 November 2022, of which 31,775 questionnaires were completed between 24 August 2021 and 14 November 2022, and therefore included solicited AE data on injection site and systemic AE (Table 1). For 91.6% (30,898/33,742) of vaccines reported during the study, an AE questionnaire was completed.

**Table 1:** Number of questionnaires completed and participants included for each research objective, the Netherlands, 3 May 2021–14 November 2022

Categories	Determinants of AE			Pre-vaccination serology			Post-vaccination serology		
	Questionnaires (n = 47,947)		Participants (n = 28,032)	Questionnaires (n = 10,915)		Participants (n = 10,183)	Questionnaires (n = 2,543)		Participants^a^ (n = 2,515)
	n	%	n	n	%	n	n	%	n
Age group (years)									
18–59	23,082	48.1	14,084	6,002	55	5,719	1,206	47.4	1,197
60–85	24,830	51.8	13,920	4,910	45	4,461	1,336	52.5	1,317
Missing	35	0.1	28	3	0.0	3	1	0.0	1
Gender									
Man	17,047	35.6	10,060	3,844	35.2	3,599	844	33.2	836
Woman	30,869	64.4	17,955	7,071	64.8	6,584	1,699	66.8	1,679
Other	31	0.1	17	NA			NA		
Medical risk group^b^									
No	32,997	68.8	19,670	7,825	71.7	7,317	1,827	71.8	1,809
Yes	14,950	31.2	8,362	3,090	28.3	2,866	716	28.2	706
Vaccine product^c^									
Comirnaty	22,698	47.3	16,385	4,878	44.7	4,735	1,381	54.3	1,371
JCovden	848	1.8	846	NA			NA		
Spikevax	19,147	39.9	14,482	5,949	54.5	5,740	1,144	45.0	1,139
Vaxzevria	3,231	6.7	2,582	NA			NA		
Missing	2,023	4.2	1,973	88	0.8	86	18	0.7	18
Vaccine dose^c^									
1	9,121	19.0	9,121	NA			NA		
2	10,963	22.9	10,963	2,518	23.1	2,518	701	27.6	701
3	15,601	32.5	15,601	6,981	64.0	6,981	1,126	44.3	1,126
4	8,779	18.3	8,779	1,416	13.0	1,416	716	28.2	716
5	2,219	4.6	2,219	NA			NA		
Missing	1,264	2.6	1,264	0	0.0	0	0	0.0	0

AE: adverse events.

^a^ Participants selected for the third analysis were all COVID-naïve.

^b^ Participants were categorised as belonging to a medical risk group if they had one or more of the following conditions: asplenia, cancer (in the past, currently treated, currently untreated), cardiovascular disease, diabetes, immune disorder, kidney disease, liver disease, lung disease or asthma, neurological disease, organ or bone marrow transplantation.

^c^ COVID-19 vaccination was initiated on 6 January 2021 with Comirnaty, Spikevax, Vaxzevria and JCovden. Vaxzevria was no longer used after November 2021. Nuvaxovid was offered from March 2022 onwards. JCovden, Vaxzevria doses were excluded from the analyses evaluating pre- and post-vaccination serology data and Nuvaxovid from all analyses because of low numbers. For each research question, a selection of participants was included, who could contribute more than one dose or vaccine product, therefore numbers in the vaccine product and vaccine dose rows may add up to more than the participant number at the top (number of unique participants included).

The association between pre-vaccination S-antibody levels and AE occurrence was assessed using 10,918 questionnaires, completed by 10,183 participants, for which a blood sample within 8 weeks before vaccination was available. Eleven participants were excluded because they reported infection between blood sampling and subsequent vaccination. The relationship between AE and post-vaccination S-antibody response was assessed using 2,543 AE questionnaires, completed by 2,515 SARS-CoV-2-naive participants, for which a blood sample between 3 and 8 weeks after vaccination was available. Overall, more women than men were included, and the majority of participants received Comirnaty or Spikevax (Table 1).

### Solicited adverse events and adverse events for which medical care was sought

Injection site AE were reported in 42.4% of questionnaires and systemic AE in 33.8% of questionnaires (Table 2). Questionnaires completed by participants aged 18–59 years reported more injection site AE (49.3% vs 36.9%; p < 0.001) and systemic AE (41.8% vs 27.3%; p < 0.001) than questionnaires by participants aged 60–85 years. Questionnaires completed by women reported more injection site AE (50.0% vs 28.8%; p < 0.001) and systemic AE (38.4% vs 25.5%; p < 0.001) than questionnaires by men.

**Table 2:** Reported adverse events that were solicited (n = 31,775 questionnaires) or for which medical care was sought (n = 47,947 questionnaires), the Netherlands, 3 May 2021–14 November 2022

Categories	Solicited AE (n = 31,775 questionnaires)							AE for which medical care was sought (n = 47,947 questionnaires)		
	Injection site AE			Systemic AE						
	Number of events	Number of questionnaires	%	Number of events	Number of questionnaires	%	Number of events		Number of questionnaires	%
Reported AE	13,479	31,775	42.4	10,725	31,775	33.8	1,034		47,947	2.2
Age group (years)										
18–59	6,950	14,105	49.3	5,897	14,105	41.8	586		23,082	2.5
60–85	6,521	17,652	36.9	4,826	17,652	27.3	446		24,830	1.8
Missing	8	18	44.4	2	18	11.1	2		35	5.7
Gender										
Men	3,275	11,356	28.8	2,893	11,356	25.5	252		17,047	1.5
Women	10,197	20,401	50.0	7,824	20,401	38.4	780		30,869	2.5
Other	7	18	38.9	8	18	44.4	2		31	6.5
Medical risk group^a^										
No	9,275	21,750	42.6	7,520	21,750	34.6	611		32,997	1.9
Yes	4,204	10,025	41.9	3,205	10,025	32.0	423		14,950	2.8
Vaccine product^b^										
Comirnaty	5,499	12,595	43.7	4,448	12,595	35.3	494		22,698	2.2
Jcovden	21	70	30.0	30	70	42.9	23		848	2.7
Spikevax	7,494	17,589	42.6	5,825	17,589	33.1	336		19,147	1.8
Vaxzevria	51	199	25.6	37	199	18.6	137		3,231	4.2
Missing	414	1,322	31.3	385	1,322	29.1	44		2,023	2.2
Vaccine dose										
1	137	259	52.9	120	259	46.3	318		9,121	3.5
2	1,879	4,104	45.8	1,711	4,104	41.7	266		10,963	2.4
3	7,324	15,568	47.0	5,682	15,568	36.5	287		15,601	1.8
4	3,144	8,779	35.8	2,384	8,779	27.2	100		8,779	1.1
5	727	2,219	32.8	569	2,219	25.6	32		2,219	1.4
Missing	268	846	31.7	259	846	30.6	31		1,264	2.5

AE: adverse events.

^a^ Participants were categorised as belonging to a medical risk group if they had one or more of the following conditions: asplenia, cancer (in the past, currently treated, currently untreated), cardiovascular disease, diabetes, immune disorder, kidney disease, liver disease, lung disease or asthma, neurological disease, organ or bone marrow transplantation.

^b^ COVID-19 vaccination was initiated on 6 January 2021 with Comirnaty, Spikevax, Vaxzevria and JCovden. Vaxzevria was no longer used after November 2021. Nuvaxovid was offered from March 2022 onwards. JCovden and Vaxzevria doses were excluded from the analyses evaluating pre- and post-vaccination serology data and Nuvaxovid from all analyses because of low numbers.

Injection site AE were reported most often after vaccination with Comirnaty (43.7%) and systemic AE after vaccination with Jcovden (42.9%). Injection site AE ranged between 52.9% after the first dose and 32.8% after the fifth dose. The percentage of participants experiencing systemic AE decreased after each dose from 46.3% after the first dose to 25.6% after the fifth dose. However, this trend disappeared when data were stratified by age group (Supplementary file 3 Figure S1 and S2, providing frequencies of occurrence, duration and severity of systemic and injection site AE). The median duration of injection site AE was 2 days and median severity was 3 on a 10-point Likert scale. For systemic AE, the median duration was 2 days and median severity was 4 on a 10-point Likert scale.

In 1,034 questionnaires (2.2%), participants reported seeking medical care for a potential AE, of which the majority were women (75%; 780/1,034) and aged 18–59 years (57%). Adverse events for which medical care was sought were reported most often after Vaxzevria (4.2%) and after the first dose (3.5%) (Table 2).

Logistic regression analysis including only mRNA vaccines showed that younger age (18–59 years), being a woman and belonging to a medical risk group were associated with higher frequency of reporting injection site AE, systemic AE and AE for which medical care was sought (Table 3). Additionally, receiving Spikevax was associated with higher frequency of injection site AE (odds ratio (OR): 1.30; 95% CI: 1.23–1.37) and systemic AE (OR: 1.28; 95% CI: 1.21–1.35) compared with Comirnaty. The odds of reporting injection site AE, systemic AE or AE for which medical care was sought decreased between the first and fifth doses.

**Table 3:** Multivariable logistic regression analysis estimating determinants for occurrence of adverse events after mRNA COVID-19 vaccination, the Netherlands, 3 May 2021–14 November 2022

Categories	Solicited AE (n = 30,145)				AE for which medical care was sought (n = 41,937)	
	Injection site AE		Systemic AE			
	OR	95% CI	OR	95% CI	OR	95% CI
Reported AE (n)	12,979		10,265		828	
Age group (years)						
18–59	Reference					
60–85	0.70	0.66–0.74	0.59	0.55–0.63	0.70	0.59–0.83
Gender^a^						
Man	Reference					
Woman	2.39	2.26–2.53	1.71	1.61–1.81	1,77	1.49–2.10
Medical risk group^b^						
No	Reference					
Yes	1.13	1.07–1.20	1.07	1.01–1.14	1.75	1.51–2.04
Vaccine product						
Comirnaty	Reference					
Spikevax	1.30	1.23–1.37	1.28	1.21–1.35	1.14	0.97–1.34
Vaccine dose						
Dose 1	Reference					
Dose 2	0.59	0.44–0.79	0.79	0.60–1.05	0.71	0.59–0.86
Dose 3	0.67	0.50–0.89	0.73	0.55–0.97	0.58	0.48–0.71
Dose 4	0.46	0.35–0.62	0.56	0.42–0.74	0.38	0.29–0.49
Dose 5^c^	0.42	0.31–0.57	0.55	0.41–0.74	0.49	0.33–0.75

AE: adverse events; CI: confidence interval; OR: odds ratio.

^a^ Gender ‘other’ was excluded from the analysis because of low numbers.

^b^ Participants were categorised as belonging to a medical risk group if they had one or more of the following conditions: asplenia, cancer (in the past, currently treated, currently untreated), cardiovascular disease, diabetes, immune disorder, kidney disease, liver disease, lung disease or asthma, neurological disease, organ or bone marrow transplantation.

^c^ Less than 5% of participants who received Dose 5 at the time of this study were under 60 years of age.

Participants with certain underlying conditions or medication use more often reported AE compared with those without, and the frequency and type of reported AE also differed by age group. Supplementary file 3 Figures S3 and S4 provides proportions of occurrence of adverse events by medical conditions and medication use. More specifically, in both age groups, participants with gastrointestinal disease, lung disease or asthma, and those who used gastroprotective medication reported more injection site and systemic AE than those without underlying conditions or medication use. Participants in both age groups with gastrointestinal disease, lung disease or asthma, and participants who used immunosuppressive medication reported more AE for which medical care was sought (Supplementary file 3 Figures S3 and S4).

The three most frequently reported AE for which medical care was sought were headache, fatigue and fever. The top 20 of most frequently reported AE are provided in Supplementary file 3 Figure S5.

### Pre-vaccination S-antibody concentrations and adverse events

**Figure 1:**
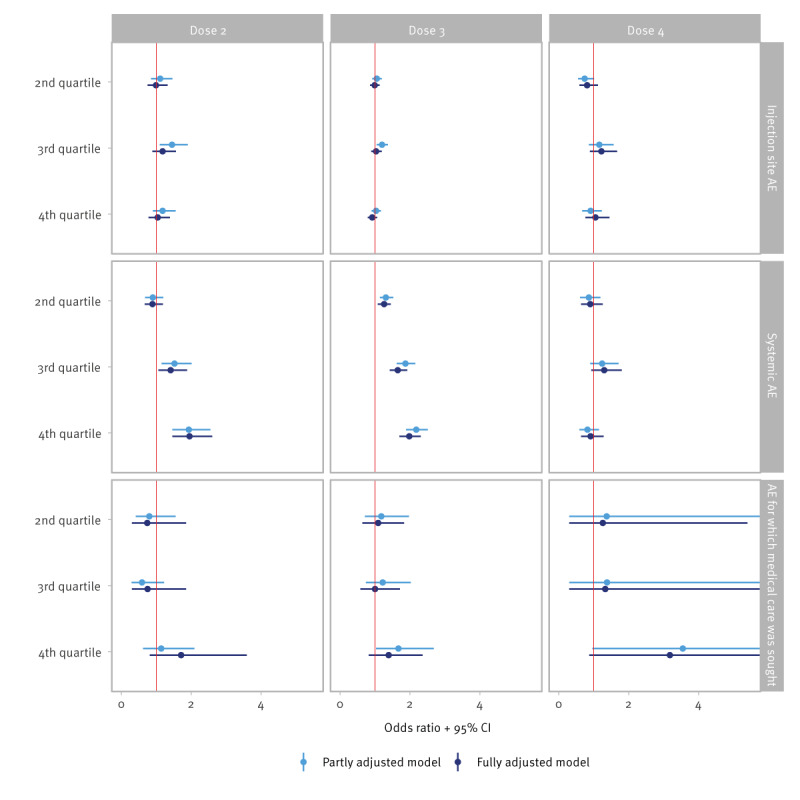
Association between pre-vaccination SARS-CoV-2 S-antibody concentrations in quartiles^a^ and type of adverse events stratified by COVID-19 vaccine dose, the Netherlands, 3 May 2021–14 November 2022 (n = 10,915 vaccinations)

Logistic regression showed that the odds for experiencing systemic AE after Dose 2 was significantly increased for those with pre-vaccination S-antibody concentrations in the third (fully adjusted OR: 1.41; 95% CI: 1.06–1.88) and fourth (OR: 1.95; 95% CI: 1.46–2.60) quartiles compared with those in the lowest quartile (Figure 1 and Supplementary File S4 for the partly and fully adjusted ORs). The odds of experiencing systemic AE after Dose 3 increased with increasing pre-vaccination S-antibody concentrations; the fully adjusted OR increased from 1.26 (95% CI: 1.08–1.45) in the second quartile to 1.98 (95% CI: 1.70–2.31) in the fourth quartile. For systemic AE after Dose 4, no association was observed with pre-vaccination concentrations. Some statistically significant associations were observed for pre-vaccination S-antibody concentrations and injection site AE and AE for which medical care was sought in the partly adjusted model, but results were not statistically significant when fully adjusted for time between blood sampling and vaccination, vaccine product, gender and age.

Pre-vaccination S-antibody concentrations are measured in binding antibody units (BAU)/ml. Partly adjusted models were adjusted for time between fingerprick and vaccination. Fully adjusted models were adjusted for time between fingerprick and vaccination, vaccine product, age and gender.

^a^ Quartiles per dose. Dose 2: 20–151–452–1,502–142,096; Dose 3: 20–375–933– 2,209–225,000; Dose 4: 24–7,321–19,997–44,335–225,000.

### Adverse events and post-vaccination SARS-CoV-2 S-antibody concentrations

Geometric mean concentrations post-vaccination were higher in participants with AE compared with those without AE (5,438 vs 4,022 BAU/ml after Dose 2; 20,510 vs 15,553 BAU/ml after Dose 3; 23,581 vs 20,948 BAU/ml after Dose 4).

**Figure 2:**
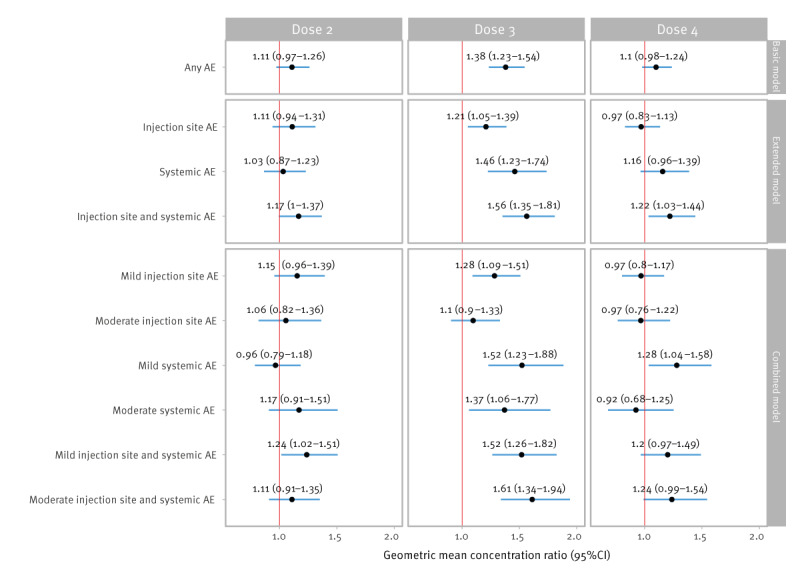
Geometric mean concentration ratios of post-vaccination SARS-CoV-2 S-antibody concentrations for any AE, type of AE, and type and severity of AE, the Netherlands, 3 May 2021–14 November 2022

After Dose 2, a statistically significant GMC ratio of 1.24 (95% CI: 1.02–1.51; Figure 2) was observed for occurrence of mild injection site and systemic AE (combined model) compared with no AE. There were no other significant associations.

Models were adjusted for age group, gender, medical risk group, body mass index, vaccine product and time between blood sampling and vaccination. Analysis was restricted to participants having received a mRNA vaccine as Dose 2 or 3, and participants aged 60 years or older having received a mRNA vaccine as Dose 4.

Occurrence of any AE after Dose 3 (basic model) was associated with significantly higher antibody concentrations post-vaccination (GMC ratio: 1.38; 95% CI 1.23–1.54). Compared with experiencing no AE, the highest GMC ratio was observed for those experiencing both injection site and systemic AE (GMC ratio: 1.56; 95% CI: 1.35–1.81). In the combined model, estimated differences in S-antibody concentrations were highest for those experiencing moderate injection site and systemic AE (GMC ratio: 1.61; 95% CI: 1.34–1.94) compared with no AE.

After Dose 4, experiencing both injection site and systemic AE (GMC ratio: 1.22; 95% CI: 1.03–1.44) and mild systemic AE (GMC ratio: 1.28; 95% CI: 1.04–1.58) were associated with higher S-antibody concentrations.

## Discussion

This study aimed to investigate solicited AE and AE for which medical care was sought after COVID-19 vaccination in the Netherlands and to determine the relationship between self-reported AE and anti-SARS-CoV-2 antibody concentrations before and after mRNA vaccination.

In this study, injection site and systemic AE were reported after any COVID-19 vaccination in ca 42 and 34% of questionnaires, respectively. Our results on frequency, severity and determinants of solicited – injection site and systemic – AE are substantiated by other studies [5,21]. Rolfes et al. performed a cohort event monitoring study in the Netherlands and found that, compared with Comirnaty, the JCovden, Vaxzevria and Spikevax vaccines were associated with increased reporting of reactogenicity [5]. In line with our findings, they also found increased reporting of AE in women and younger participants. Differences in AE reporting between specific groups may be due to differences in the immune response according to age and sex [7]. The immune system declines with age in the process of immune senescence; vaccination elicits a weaker immune response and therefore possibly resulting in less AE in older age groups [7]. Furthermore, while differences in AE reporting may be due to differences in reporting behaviour between genders, there are biological explanations for differences in AE reporting, e.g. the immunosuppressive properties of testosterone compared with oestrogen influence COVID-19 vaccination response and therefore reactogenicity according to sex [7].

Rolfes et al. also found increased reporting of AE for several medical conditions, consistent with our findings. For some conditions, such as lung disease or asthma and cancer, an increased reporting might be explained by a higher risk for symptoms such as fever in general [5]. Increased reporting might also result from more frequent contact with HCP in general or increased alertness for medical occurrences compared with the population without medical conditions. Importantly, as those with underlying conditions are at higher risk for severe COVID-19 disease, vaccination is crucial. Therefore, maintaining vaccine acceptance is especially important in this group; their ongoing contact with the healthcare system should be used to disseminate accurate information on AE and emphasise the importance of vaccination [22].

Our findings on AE for which medical care was sought are consistent with previous findings by a prospective cohort study performed in Australia [23]. In our study, headache, fatigue and fever were the most commonly reported AE. These AE are listed in the Summary of Product Characteristics (SmPC) of the Comirnaty and Spikevax vaccines [24,25]. These are generally mild and expected AE, but our study found that they may cause vaccinees to seek medical attention, possibly in relation to younger age and underlying conditions or medication use. Additionally, extensive (social) media attention regarding the risk of cerebral venous thrombosis in relation to the use of Vaxzevria may have also impacted health-seeking behaviour after experiencing headache [26].

Notably, some studies report an increase in AE after the second and third dose of COVID-19 vaccination as compared with the first dose [21,27]. We had limited data on injection site and systemic AE after the first dose and therefore we could not validly study this. However, compared with the first dose, systemic AE seemed actually to decrease after subsequent doses in our study. This could be due to decreased vaccine uptake among those who experienced a higher burden of AE after their first vaccination.

Increased AE after the second and booster doses might be due to pre-existing immunity resulting from previous vaccine doses [7]. Antibody levels prior to a vaccination are influenced by the interval between previously administered vaccines or infection and vaccination, with longer dosing intervals between vaccinations possibly leading to higher antibody responses [28]. Additionally, previous research has shown that those receiving heterologous vaccination compared with those receiving homologous vaccination had slower waning of antibodies [29]. As vaccine scheduling in regard to both dosing and vaccine product administered differed between countries [30], this might also explain why some studies reported an increase in AE after the second and third dose. In our study, there was an association between antibody concentrations before vaccination and systemic AE after the second and third vaccine doses. Previous research on the relationship between antibody levels just before vaccination and AE has conflicting results [7] and more research is needed to uncover this mechanism.

Regarding the relationship between AE and post-mRNA-vaccination antibody response, some studies using comparable methods found an association between second dose mRNA vaccine AE and post-vaccination S-antibody concentrations, in contrast to our study [9,11,12,31,32]. Other research, however, is in alignment with our findings and did not find a correlation or no strong enough evidence to conclude that AE occurrence reflects higher antibody response after the second dose [13,14]. There is limited research on AE after booster COVID-19 vaccination and the relationship with antibody levels. A study from September 2022 on the Comirnaty first booster reported a trend for higher antibodies in the month after vaccination in participants who experienced fever, but this association was not significant [33]. 

The mechanisms in which vaccination induces the immune response are also known to be involved with systemic side-effects, such as fever [7], although no signification association was found in our study. Therefore, an association between AE and antibody response may be expected. A study on the persistence of SARS-CoV-2 antibodies related to symptoms found that those with COVID-19-like symptoms had higher antibody concentrations for IgG and IgM compared with those who were asymptomatic or had only mild symptoms, possibly because of a stronger inflammatory response [34].

In our study, we mainly found associations between AE and both pre- and post-mRNA-vaccination antibody levels after the third mRNA vaccine dose. Previous research has shown increasing post-vaccination anti-S antibody concentrations for subsequent vaccine doses, with maximal immunogenicity reached after the third dose [15,35]. Two clinical trials investigating the immunogenicity of fourth mRNA vaccine doses found that participants with high levels of response before the fourth dose only had limited boosting after the fourth vaccination because of a ceiling effect; previous antibody levels are restored, but do not surpass immunogenicity reached after the third dose [35,36]. This may explain why we observed no association or weaker associations between AE and pre- and post-mRNA-vaccination antibody levels, respectively, after the fourth dose. Variation between doses may also originate from adaptations made to COVID-19 vaccines to target new SARS-CoV-2 variants. Additionally, the concentration of the Spikevax booster dose was halved, possibly leading to different antibody trajectories and AE occurrence compared with the full dose administered in the primary series [37].

Across studies, the mixed results on the relationship between AE and antibody response may result from differences in methodology. For example, we collected data on injection site and systemic AE overall, but S-antibody acquisition may instead be reflected by specific injection site or systemic AE. Kobashi and colleagues found a correlation with muscle and joint pain, and fever after a second dose mRNA vaccination, but not with fatigue or headache [38]. Disparity between studies may also originate from varying antibody trajectories resulting from differences in vaccine schedules and vaccine products [28]. The timepoint of blood sample measurement also differed across studies. Oyebanji et al., Uwamino et al. and Braun et al. collected blood samples at approximately 2 weeks, 3 weeks and 1 month after the second dose respectively, whereas we included blood samples taken between 3 to 8 weeks after vaccination [9,12,27]. In addition, many studies were performed in HCP populations as opposed to the general population, but HCP were found to have higher IgG peak levels after vaccination compared with non-HCP, potentially because of the healthy worker effect [28]. Moreover, a wide variety of assays was used to assess different serology outcome measures. These methodological differences decrease comparability of studies.

A strength of this study is the large study population for which extensive information was available on covariables. Participants with medical risk conditions are oversampled allowing sufficient numbers to study this group. In general, VASCO participants are more often women, highly educated and of Dutch ethnicity compared to the overall population in the Netherlands [16]. Furthermore, no persons under the age of 18 years or persons living in an institution were included, reducing representativeness in these populations. However, we were able to exclude participants with a history of SARS-CoV-2 infection, which is associated with both increased reactogenicity and immunogenicity, from our analyses on the relationship between AE occurrence and antibody response [39]. In addition, we included vaccinations beyond the second vaccination dose in our analyses, which contributes to the applicability of this study. This study also has several limitations. Firstly, AE questionnaires could be subject to recall bias, which may have caused participants to under- or overestimate their experience. However, as questionnaires were made available within a relatively short timespan of 4 weeks after vaccination, bias is most likely limited. Secondly, while we were able to adjust for several variables, there were also possible confounders on which no information was available. For example, we were not able adjust for the (daily) use of antipyretic drugs, which has been linked to blunted SARS-CoV-2 antibody responses [7,38]. Not considering antipyretic drug use in our analyses may have overestimated the association between AE and antibody response. Thirdly, the association between antibody response and AE may be confounded by pre-vaccination antibody levels. In this study, we found an association between pre-vaccination levels and the occurrence of AE after vaccination. Because of low numbers of participants with a blood sample shortly before and shortly after vaccination, we could not adjust for pre-vaccination antibody levels in our analysis of the relationship between AE and post-vaccination antibody levels, which may have influenced our findings.

## Conclusions

This study showed that, for up to 43% of vaccine doses, AE were reported shortly after COVID-19 vaccination. Being a woman, younger age (18–59 yrs), belonging to a medical risk group and receiving Spikevax compared with Comirnaty were associated with experiencing AE. Higher pre-vaccination S-antibody levels were also associated with experiencing systemic AE after the second and third mRNA COVID-19 vaccine doses. In addition, experiencing AE may reflect higher antibody acquisition, as we observed after booster doses. Our research provides evidence regarding determinants for AE, including the role of pre-vaccination antibody levels, as well as evidence for the association between AE and COVID-19 vaccination antibody response.

## Supplementary Data

1053000 Supplement
