# Supplementary material for: Association between adverse events after COVID-19 vaccination and anti-SARS-CoV-2 antibody concentrations, the Netherlands, May 2021 to November 2022: a population-based prospective cohort study
Source: Euro Surveill. 2024 Jun 20;29(25):2300585. doi: 10.2807/1560-7917.ES.2024.29.25.2300585 (PMC11191418; doi:10.2807/1560-7917.ES.2024.29.25.2300585)
Supplement: Supplement [file 23-00585_KNOL_Supplement.pdf]

Supplementary file 1 – Sampling moments in VASCO

This supplementary material is hosted by *Eurosurveillance* as supporting information alongside the article Association between adverse events after COVID-19 vaccination and anti-SARS-CoV-2 antibody concentrations, the Netherlands, May 2021 to November 2022: a population-based prospective cohort study, on behalf of the authors, who remain responsible for the accuracy and appropriateness of the content. The same standards for ethics, copyright, attributions and permissions as for the article apply. Supplements are not edited by *Eurosurveillance* and the journal is not responsible for the maintenance of any links or email addresses provided therein.

Supplementary figure S1. Timeline example participants

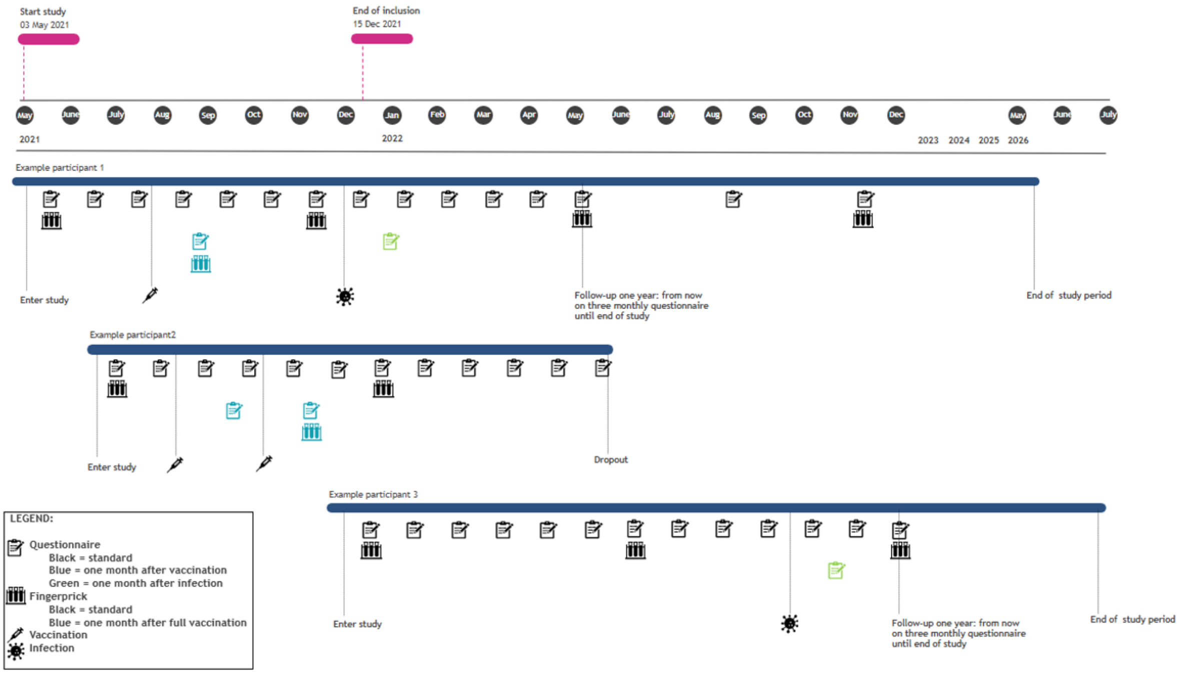

Source: Huiberts AJ, Kooijman MN, Melker HEd, Hahne SJ, Grobbee DE, Hoeve C, et al. Design and baseline description of an observational population-based cohort study on COVID-19 vaccine effectiveness in the Netherlands - The VAccine Study COvid-19 (VASCO). Research Square. 2022 May 19. https://doi.org/10.21203/rs.3.rs-1645696/v1

Supplementary file 2 – Questionnaire 1 month after COVID-19 vaccination

This supplementary material is hosted by *Eurosurveillance* as supporting information alongside the article Association between adverse events after COVID-19 vaccination and anti-SARS-CoV-2 antibody concentrations, the Netherlands, May 2021 to November 2022: a population-based prospective cohort study, on behalf of the authors, who remain responsible for the accuracy and appropriateness of the content. The same standards for ethics, copyright, attributions and permissions as for the article apply. Supplements are not edited by *Eurosurveillance* and the journal is not responsible for the maintenance of any links or email addresses provided therein.

Questions 7-9 were included from May 2021 till end of study period, questions 1-6 were added from August 2021.

Questions:

You have been vaccinated against coronavirus approximately a month ago.

1. Did you experience symptoms around the administration site within one week after vaccination? For example, redness, pain, or swelling around the administration site or underarm.

- No
- Yes

If ‘yes’ to question 1:

2. how severe did you consider these symptoms? 1 means very mild symptoms, 10 means very severe symptoms

Very mild symptoms									Very severe symptoms
1	2	3	4	5	6	7	8	9	10

3. How long did these symptoms last?

- <1 day
- 1 – 2 days
- 3 – 4 days
- 5 or more days

4. Did you experience other symptoms within one week after vaccination? For example, fever, headache, myalgia, tiredness, nausea, diarrhea, or other symptoms of malaise/feeling unwell.

- No
- Yes

If ‘yes’ to question 4:

5. How severe did you consider these symptoms? 1 means very mild symptoms, 10 means very severe symptoms

Very mild symptoms									Very severe symptoms
1	2	3	4	5	6	7	8	9	10

6. How long did these symptoms last?

- <1 day
- 1 – 2 days
- 3 – 4 days
- 5 or more days

7. Were you in contact with your GP or other healthcare provider in the last month due to a possible adverse event of the COVID-19 vaccine?

- No
- Yes

If ‘yes’ to question 7:

8. For which symptoms did you contact your GP or other healthcare provider?

[open text field]

9. Do you have any comments with regards to this questionnaire? If not, this field may be left empty.

[open text field]

Supplementary file 3 – solicited AE and AE for which medical care was sought

This supplementary material is hosted by *Eurosurveillance* as supporting information alongside the article Association between adverse events after COVID-19 vaccination and anti-SARS-CoV-2 antibody concentrations, the Netherlands, May 2021 to November 2022: a population-based prospective cohort study, on behalf of the authors, who remain responsible for the accuracy and appropriateness of the content. The same standards for ethics, copyright, attributions and permissions as for the article apply. Supplements are not edited by *Eurosurveillance* and the journal is not responsible for the maintenance of any links or email addresses provided therein.

Supplementary Figure S3.1. Injection site AE occurrence (yes/no), duration (days) and severity (10-point likert scale) by age group

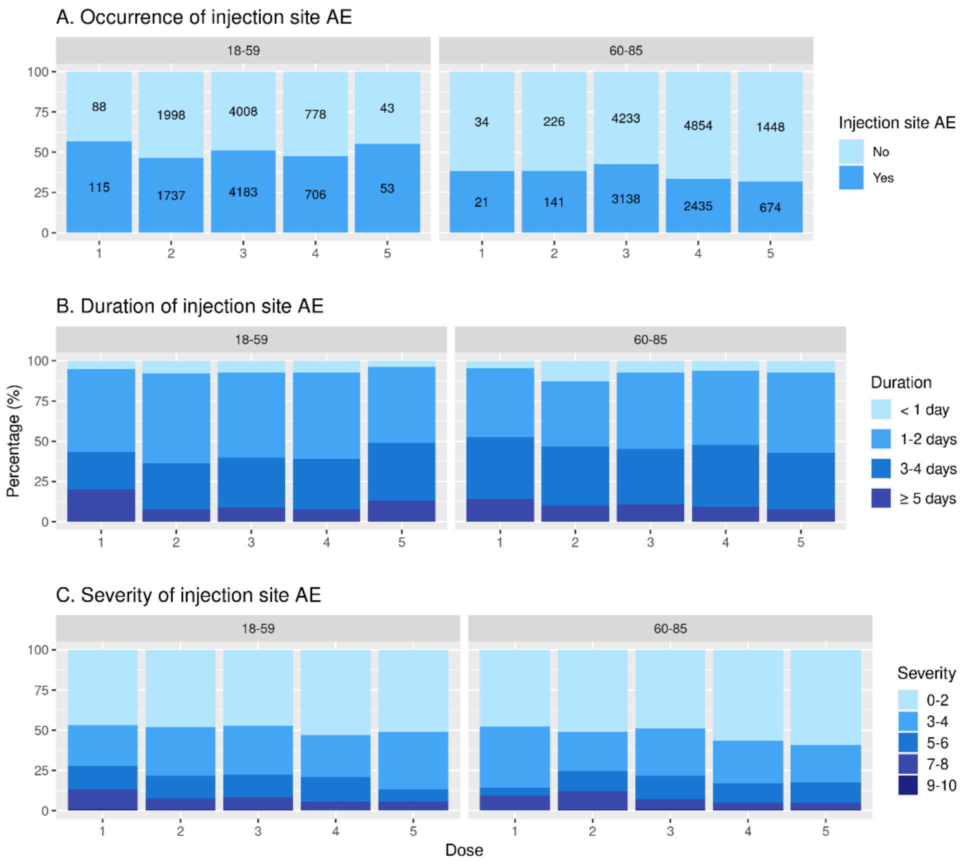

Supplementary Figure S3.2. Systemic AE occurrence (yes/no), duration (days) and severity (10-point likert scale) by age group

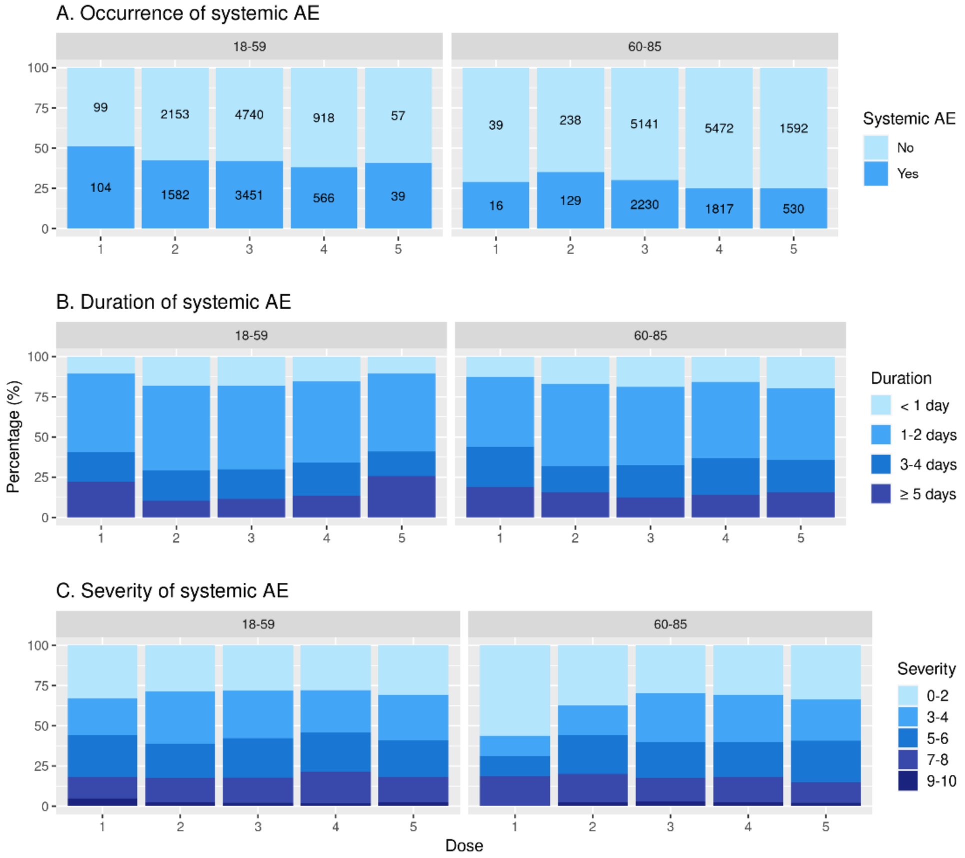

Supplementary Figure S3.3. Injection site AE, systemic AE and AE for which medical care was sought reporting by underlying condition

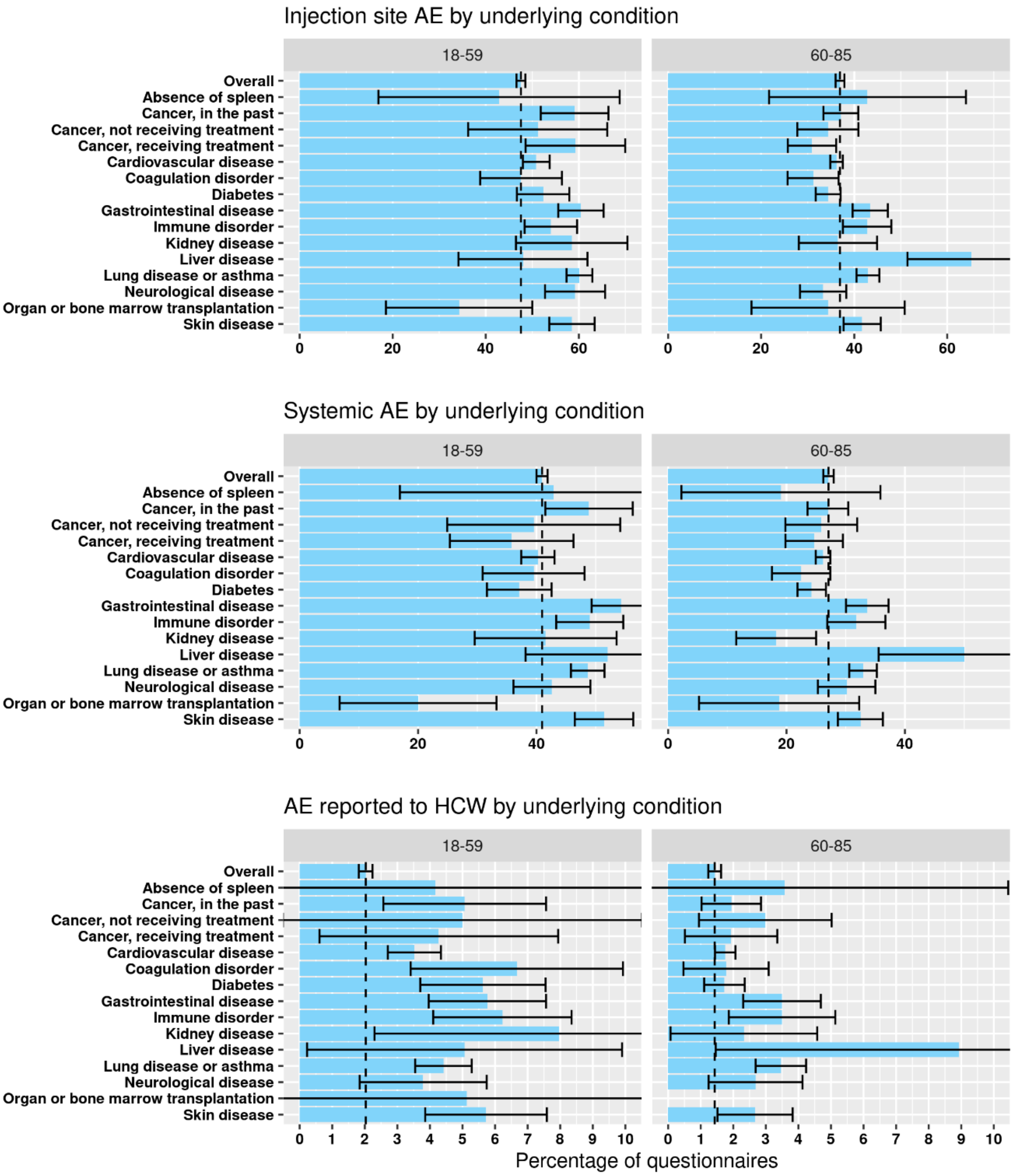

Supplementary figure S3.4. Injection site AE, systemic AE and AE for which medical care was sought reporting by medication use

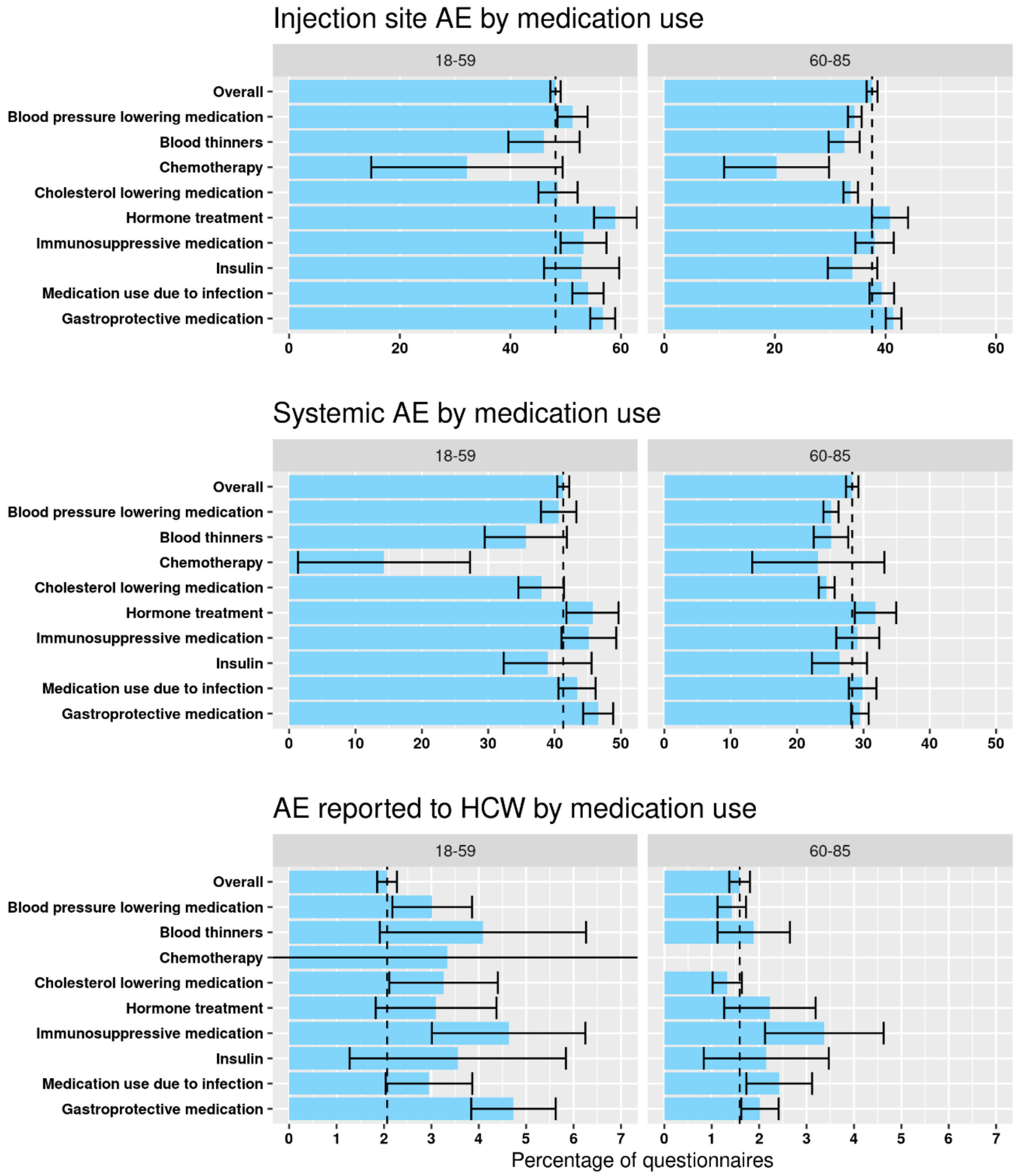

Supplementary Figure S3.5. Top 20 most frequently reported AE for which medical care was sought

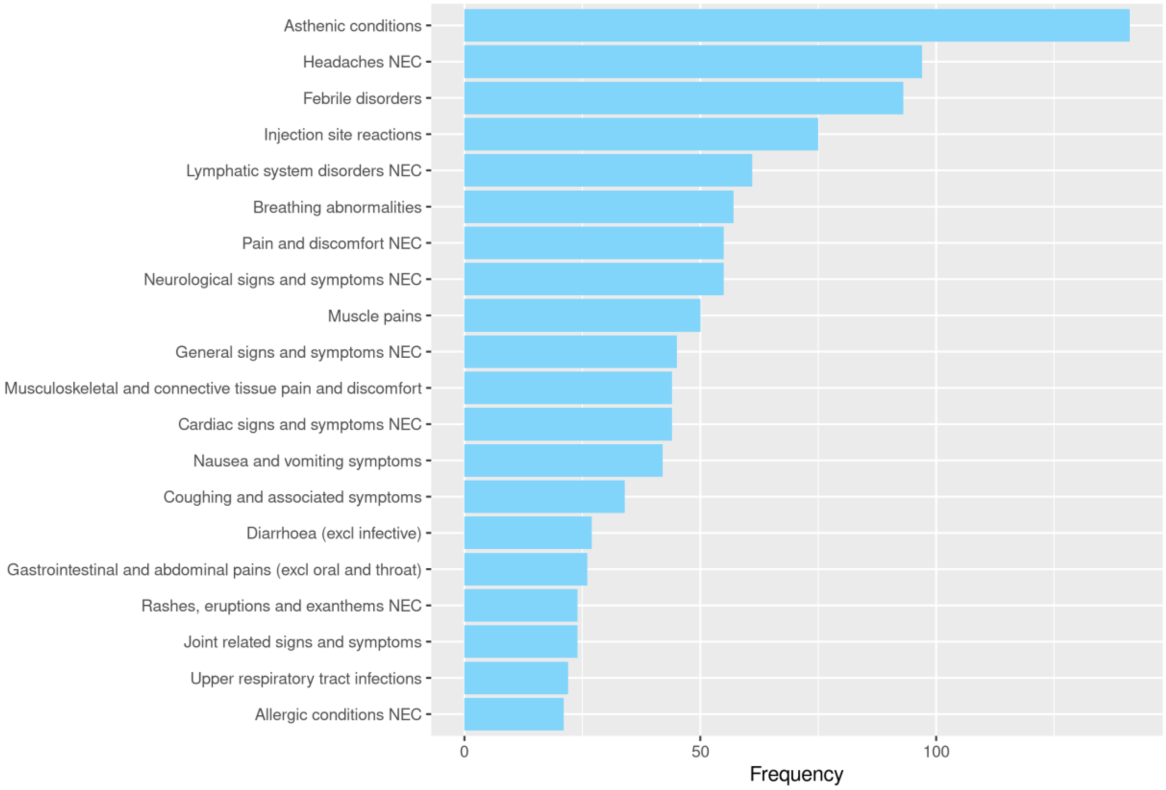

Supplementary file 4. Pre-vaccination S-antibody concentrations and AE

This supplementary material is hosted by *Eurosurveillance* as supporting information alongside the article Association between adverse events after COVID-19 vaccination and anti-SARS-CoV-2 antibody concentrations, the Netherlands, May 2021 to November 2022: a population-based prospective cohort study, on behalf of the authors, who remain responsible for the accuracy and appropriateness of the content. The same standards for ethics, copyright, attributions and permissions as for the article apply. Supplements are not edited by *Eurosurveillance* and the journal is not responsible for the maintenance of any links or email addresses provided therein.

Supplementary figure S4.1. Partly and fully adjusted odds ratios with 95% confidence intervals for the association between pre-vaccination S-antibody concentrations (BAU/ml) in quartiles and type of AE stratified by dose.

	Injection site AE		Systemic AE		AE for which medical care was sought	
	Partly adjusted[1] OR (95% CI)	Fully adjusted[2] OR (95% CI)	Partly adjusted OR (95% CI)	Fully adjusted OR (95% CI)	Partly adjusted OR (95% CI)	Fully adjusted OR (95% CI)
**Dose 2**						
1st quartile (20 - 151)	Reference					
2nd quartile (151 – 452)	1.11 (0.85; 1.46)	0.99 (0.75; 1.32)	0.90 (0.68; 1.20)	0.89 (0.67; 1.19)	0.80 (0.41; 1.55)	0.74 (0.30; 1.85)
3rd quartile (452 – 1,502)	**1.45 (1.10; 1.90)**	1.18 (0.89; 1.56)	**1.52 (1.15; 2.01)**	**1.41 (1.06; 1.88)**	0.59 (0.29; 1.22)	0.75 (0.30; 1.85)
4th quartile (1,502 - 142,096)	1.18 (0.90; 1.55)	1.04 (0.78; 1.39)	**1.93 (1.46; 2.55)**	**1.95 (1.46; 2.60)**	1.14 (0.62; 2.09)	1.71 (0.81; 3.59)
**Dose 3**						
1st quartile (20 – 375)	Reference					
2nd quartile (375 - 933)	1.05 (0.92; 1.19)	0.99 (0.86; 1.13)	**1.31 (1.14;1.52)**	**1.26 (1.08; 1.45)**	1.18 (0.71; 1.97)	1.09 (0.64; 1.83)
3rd quartile (933 – 2209)	**1.20 (1.05; 1.37)**	1.03 (0.89; 1.19)	**1.87 (1.62; 2.15)**	**1.65 (1.42; 1.92)**	1.22 (0.74; 2.02)	1.00 (0.58; 1.71)
4th quartile (2209 - 225,000)	1.03 (0.90; 1.17)	0.92 (0.79; 1.06)	**2.18 (1.89; 2.51)**	**1.98 (1.70; 2.31)**	**1.67 (1.03; 2.68)**	1.39 (0.82; 2.36)
**Dose 4**						
1st quartile (24 – 7,321)	Reference					
2nd quartile (7321 – 19,977)	0.74 (0.55; 1.01)	0.81 (0.59; 1.12)	0.86 (0.61; 1.19)	0.90 (0.64; 1.26)	1.37 (0.30; 6.14)	1.26 (0.30; 5.41)
3rd quartile (19,977 – 44,335)	1.16 (0.86; 1.57)	1.22 (0.89; 1.67)	1.24 (0.90; 1.71)	1.30 (0.93; 1.80)	1.38 (0.30; 6.25)	1.33 (0.30; 5.80)
4th quartile (44,335– 225,000)	0.91 (0.67; 1.23)	1.05 (0.76; 1.45)	0.82 (0.59; 1.15)	0.91 (0.64; 1.28)	3.55 (0.96; 13.12)	3.18 (0.87; 11.56)

[1]Adjusted for time between fingerprick and vaccination

[2]Adjusted for time between fingerprick and vaccination, vaccine product, age and sex
